# Is too much salt harmful? Yes

**DOI:** 10.1007/s00467-019-04387-4

**Published:** 2019-11-28

**Authors:** Róbert Agócs, Dániel Sugár, Attila J. Szabó

**Affiliations:** 1 1st Department of Paediatrics, Semmelweis University, Bókay János u. 53–54, Budapest, H-1083 Hungary; 2 MTA-SE Paediatrics and Nephrology Research Group, Hungarian Academy of Sciences, Semmelweis University, Budapest, Hungary

**Keywords:** Dietary sodium, Recommendation, Salt sensitivity, Blood pressure, Hypertension, Autoimmunity

## Abstract

The contribution of high sodium intake to hypertension and to the severity of immune-mediated diseases is still being heatedly debated in medical literature and in the lay media. This review aims to demonstrate two conflicting views on the topic, with the first part citing the detrimental effects of excessive salt consumption. Sodium plays a central role in volume and blood pressure homeostasis, and the positive correlation between sodium intake and blood pressure has been extensively researched. Despite the fact that the average of global daily salt consumption exceeds recommendations of international associations, health damage from excessive salt intake is still controversial. Individual differences in salt sensitivity are in great part attributed to this contradiction. Patients suffering from certain diseases as well as other vulnerable groups—either minors or individuals of full age—exhibit more pronounced blood pressure reduction when consuming a low-sodium diet. Furthermore, findings from the last two decades give insight into the concept of extrarenal sodium storage; however, the long-term consequences of this phenomenon are lesser known. Evidence of the relationship between sodium and autoimmune diseases are cited in the review, too. Nevertheless, further clinical trials are needed to clarify their interplay. In conclusion, for salt-sensitive risk groups in the population, even stricter limits of sodium consumption should be set than for young, healthy individuals. Therefore, the question raised in the title should be rephrased as follows: “how much salt is harmful” and “for whom is elevated salt intake harmful?”

## Introduction

With salt being the principal means of preserving food prior to refrigeration, the amount of salt in foodstuff has significantly increased. Indirect evidence for the salt consumption of prehistoric people comes from investigations conducted on primates or on contemporary tribes living in their natural environment (e.g., Yanomamo Indians) [1, 2]. In ancient times, prior to access to external sources of salt (i.e., salt mines), the level of daily average salt intake was low, approximately 0.1–1.0 g/day (0.04–0.4 g sodium/day), which, throughout history, has increased to today’s 8.75–10.5 g/day (3.5–4.2 g sodium/day), with significant regional differences, in most countries well above the recommendations [3].

In industrial societies, eating habits—including salt consumption—have substantially changed over the past few decades. The sodium content of processed food is well beyond recommendations without the consumer exercising control over it. About 15% of dietary sodium intake is inherent to food and ~ 71% is added during food processing. Only the remaining fraction is added while eating and cooking [4]. Dietary sodium intake appears in different chemical forms, the most common being sodium chloride (table salt), sodium bicarbonate, and sodium glutamate—a flavor enhancer found ubiquitously in processed food.

The human body, predestined to salt conservation by the evolution, could not adapt to increased salt intake. This discrepancy is reflected in the growing number of people affected by salt-sensitive hypertension and cardiovascular (CV) diseases, putting tremendous pressure on the healthcare system [5]. Conventionally volume expansion arising from salt consumption had been explained solely by the interplay between sodium homeostasis and the extracellular space. Having seen its beneficial effects, sodium restriction has become an auxiliary therapy in a number of diseases involving volume overload (e.g., hypertension, kidney diseases, CV diseases, liver cirrhosis).

Since high salt intake has long been considered a key risk factor of hypertension, several associations, organizations have made recommendations regarding daily sodium intake. According to the proposals of the World Health Organization (WHO) and the American Heart Association for normal, healthy adults, daily sodium intake should not exceed the level of 2.0 g/day or 2.3 g/day, respectively [6, 7]. Since children have a lower need for sodium, their daily dietary sodium intake is different from that of adults. Recommended sodium intakes vary by age; the reference values from the National Academy of Medicine (USA) are listed in Table 1 [8].

**Table 1:** Sodium intake recommendation for children and adolescents made by the National Academy of Medicine (USA) [8]

Age (years)	Recommended maximum daily sodium intake (mg/day)
1–3	1500
4–8	1900
9–13	2200
14–18	2300

Despite the recommendations, health damage from excessive salt intake is still a subject of debate without the experts reaching consensus about the rate of optimal salt intake. This is partly due to the flawed design of studies and partly due to individual reactions to elevated salt intake [9]. The often inconsistent use of the terms “salt” and “sodium” in the studies concerning recommended salt intake adds to the confusion too. Therefore, distinction between salt and sodium must be made: 1 g of salt is equal to 40% of sodium and 60% of chloride.

## Sodium and blood pressure

Sodium plays a central role in human physiology: it canonically maintains and regulates extracellular body fluid volume and blood pressure (BP). Until recently local sodium handling and storage outside the kidney were not considered significant factors (as seen in the last section); only renal sodium transport and homeostasis had been described extensively. The complex interplay between sodium intake, BP, and renal sodium excretion was explored by Guyton who developed the concept of pressure natriuresis [10, 11]. According to this concept, the increased sodium intake results in an increase of extracellular volume and elevated BP that in turn induces sodium and water diuresis. When renal sodium excretion equilibrates with sodium intake, BP returns to normal. The mean arterial pressure at which exact fluid and salt balance is reached is called equilibrium point or set point. At any pressure greater than this point, net loss of salt and water occurs until the pressure returns to the equilibrium point. It is to be emphasized that the equilibrium point can shift upward when renal functions are impaired or permanently high sodium intake is provided.

The interaction of sodium intake and BP has been reflected in the work of Denton et al., which is probably the most important piece of evidence derived from animal studies [1]. Chimpanzees were placed on high-salt diet (12 g salt/day = 4.8 g sodium/day) and the animals developed hypertension, which reversed when they resumed their usual low-salt (0.25–0.5 g salt/day = 0.1–0.2 g sodium/day) diet.

Population-level studies underlie the effect of salt consumption on high BP. The INTERSALT study established relationship between the levels of salt consumption and BP at the level of individuals and at population levels. Sodium excretion and BP of 10,079 men and women aged 20–59 from 52 centers around the world were measured. Within each center, positive correlation between electrolyte excretion and BP was found and populations with a higher average dietary salt load had on average a higher rate of hypertensive patients [12, 13]. People with a higher salt consumption had a higher average BP and a greater increase of BP with age.

The relationship between dietary salt intake and BP was investigated in children, too. In 1983, Hofman et al. randomized 476 infants into normal (average sodium intake = 0.328 ± 0.124 g/day) and low salt intake (average sodium intake = 0.117 ± 0.035 g/day) groups [14]. After 25 weeks of dietary intervention, systolic BP of the low salt intake group was 2.1 mmHg (90% CI − 3.7–− 0.5; *P* < 0.05) lower than that of normal salt intake group. Fifteen years later, Geleijnse et al. reinvestigated 167 children from among the participants [15]. Results showed that low-sodium diet consumed during infancy had an effect on the participants’ BP (− 3.6 mmHg; 95% CI − 6.6–− 0.5; *P* < 0.05) at a later age, too.

Two Trials of Hypertension Prevention (TOHP) were conducted in the late 1980s and early 1990s. The aim of TOHP I was to assess the effect of several, non-pharmacological interventions (weight loss, sodium reduction, stress management, and nutritional supplements) on BP reduction. Subjects were comprised of adults with prehypertension (diastolic BP 80–89 mmHg) [16]. In sodium reduction group, sodium excretion decreased by 44 mmol/day (1.012 g sodium/day) by the end of the 18-month follow-up period. TOHP II evaluated the combined impact of weight loss and sodium reduction on a population of slightly overweight adults with prehypertension [17]. Sodium reduction alone caused an average net decrease of 40 mmol/day (0.920 g sodium/day) in urinary sodium excretion.

In each of the studies, small decreases in systolic BP/diastolic BP were seen with sodium reduction over the 18 or 36 months the trials lasted (TOHP I − 1.7/− 0.8 mmHg, *P* < 0.05; TOHP II − 1.2/− 0.7 mmHg, *P* < 0.05) [18, 19].

### Sodium and cardiovascular morbidity/mortality

As previous chapters have already suggested, individual response to high dietary sodium varies among the population. Despite this heterogeneity, in 2012, WHO recommended a maximum intake of 2 g sodium/day for the prevention of CV diseases for the general population. However, literature data regarding the positive correlation between sodium intake and the risk of CV is still controversial: some analyses even claim that neither hypertensive nor normotensive or elderly people benefit from reduced dietary sodium intake [20, 21].

In contrast, numerous publications have shown correlation between sodium intake and the risk of certain CV outcomes in the general population. In Great Britain, due to public health policy measures taken between 2003 and 2011, dietary salt consumption in the population has dropped by 1.4 g/day (0.56 g sodium/day, *P* < 0.01), contributing to the reduction in the number of ischemic heart disease and stroke-related death [22].

Two meta-analyses of prospective studies have shown correlation between higher sodium intake and the risk of stroke and CV diseases [23, 24]. Two further studies imply that sodium intake over the recommendations can increase the incidence of stroke independently of BP [25, 26]. In a prospective study from Finland, salt intake proved to be an independent risk factor for coronary heart disease, too [27]. A recent research established positive correlation between dietary salt intake and subclinical markers of CV diseases [28].

**Fig. 1:**
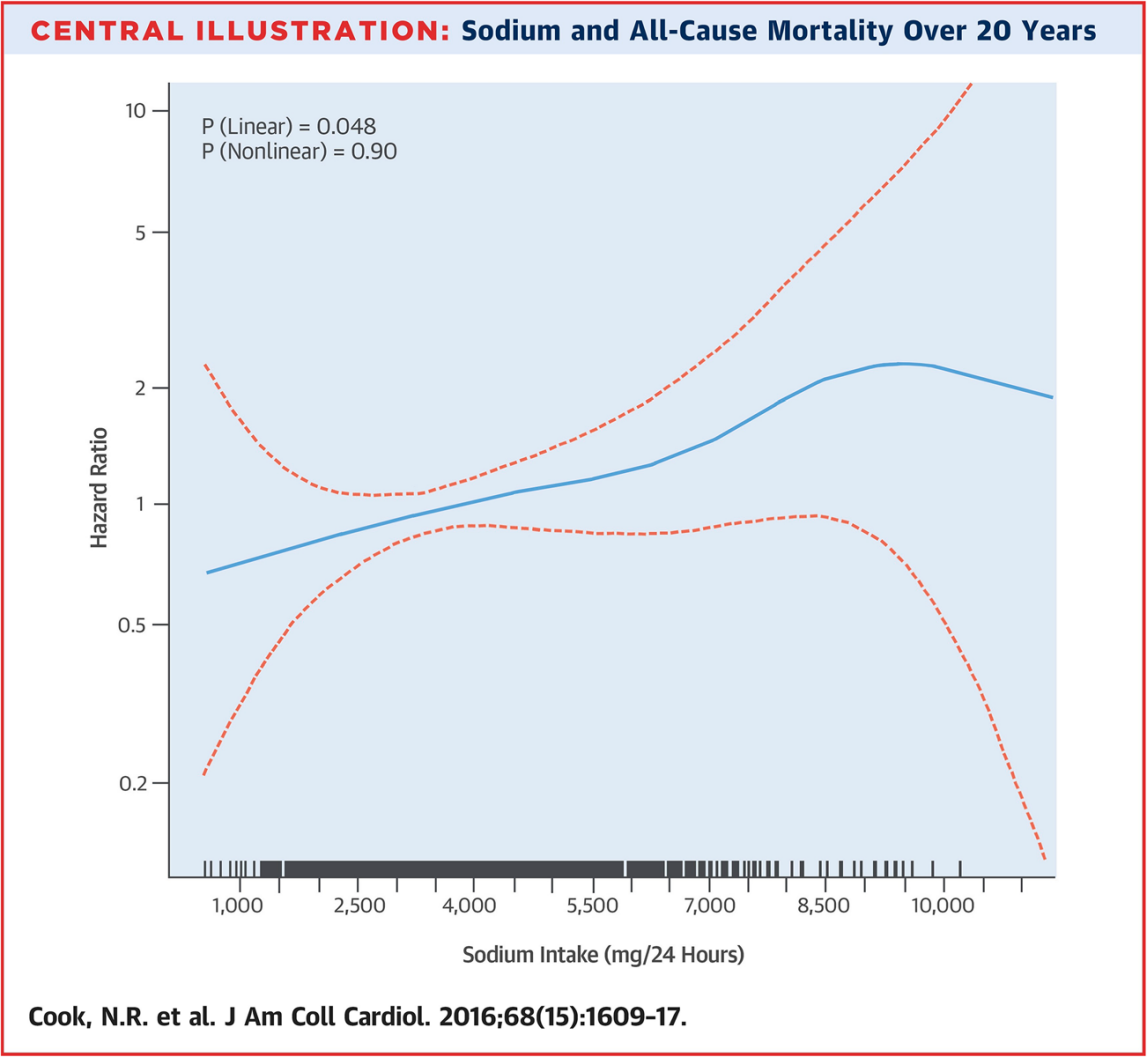
Sodium intake and all-cause mortality over 20 years

In addition to data derived from the general population, data from several special subgroups is available, too. Cook et al. followed up the prehypertensive participants of TOHP I and TOHP II for 10 or 15 years, respectively. They examined the long-term effects of sodium reduction on CV disease and mortality. At the end of the follow-up, the relative risk of CV events (heart attack, stroke, or CV-related death) was 25% lower in the sodium reduction group (relative risk = 0.75, 95% CI 0.57–0.99, *P* < 0.05). In other words, sodium intervention group had less cumulative incidence of CV disease and less cumulative mortality [29]. In 2016, after a follow-up period of 25 years, Cook et al. published data on the mortality of TOHP participants. The authors found a direct linear association between sodium intake and total mortality (*P* = 0.048) (Fig. 1) [30].

Zhao et al. conducted a prospective cohort study in China among participants with prehypertension and 30 to 70% degree of coronary artery stenosis. For participants consuming a high-salt diet (≥ 6 g salt/day = 2.4 g sodium/day), the hazard ratio was 1.97 (95% CI 1.08–2.27; *P* = 0.011) for CV diseases [31]. Among those, who consumed a high-salt diet, subgroup analysis was made. From among the subgroups, patients older than 60 years, men, overweight patients, and patients with elevated serum cholesterol level had a higher ratio for CV diseases than the group average, highlighting that these factors account for a more prominent relationship between sodium intake and CV risk. The contribution of overweight to high salt–induced hypertension is corroborated by two further studies [27, 32].

Recently, not only high-, but also low-sodium excretion has been associated with a higher CV mortality, too. This correlation showing *J*- or *U*-shaped curve has been observed in healthy individuals and in patients with type 2 diabetes [33–35].

Spline plot of average sodium intake was based on multiple 24-h excretions and total mortality in observational analysis of usual intake in the TOHP cohorts. Hazard ratios are shown for total mortality over > 20 years as a function of usual sodium intake averaged over 3 to 7 urine collections over 1 to 3 years at baseline. Mortality is lowest among those with usual sodium intake < 2300 mg/day and highest among those with levels > 6000 mg/day. There is a significant direct linear effect of sodium on mortality (*P* = 0.048) with no evidence of non-linearity (*P* = 0.90) in spline analysis (reprinted with permission from “Sodium Intake and All-Cause Mortality Over 20 Years in the Trials of Hypertension Prevention” by Cook NR et al., J Am Coll Cardiol. 2016;68(15):1609–17, [30]).

## Salt-sensitive hypertension and special risk groups in adults

Despite the data available on the association between high sodium intake and high BP, health damage from excessive salt intake is still controversial. This is attributed to individual differences in susceptibility [36].

Kempner et al. treated numerous patients suffering from severe hypertension with a strict low-salt diet (0.25 g salt/day = 0.1 g sodium/day) [37]. Consistent with previous observations, the diet markedly decreased BP and also alleviated consequences of hypertension (e.g., decreased transverse diameter of the heart on X-ray scans, and improved kidney function). This is another piece of evidence underlying that high-salt diet especially in a hypertensive population may be harmful.

Lowering sodium in the Dietary Approaches to Stop Hypertension (DASH) study had a favorable effect, too [38]. The DASH-Sodium trial studied the combined effect of different levels of dietary sodium and the DASH diet—a diet rich in vegetables, fruits, and low-fat dairy products—on hypertensive or normotensive individuals. A total of 412 participants were randomly assigned to eat either a typical American diet or a DASH diet. Sodium content within each dietary regime was either low, intermediate, or high (50, 100, 150 mmol sodium/day = 1.15, 2.30, 3.45 g sodium/day). After 30 days the trial lasted, lower sodium intake correlated with lower systolic BP in the American diet group. The most interesting finding is that mostly hypertensive patients had the highest decrease in BP following dietary salt reduction.

In a meta-analysis of randomized, controlled trials, He et al. showed that a longer term modest salt reduction trial resulted in a minimal reduction of systolic and diastolic BP in normotensive patients [39]. However, in hypertensive patients, the same level of dietary salt reduction resulted in more pronounced systolic and diastolic BP reduction.

In an important study published by Mente et al., the association of estimated intake of sodium and potassium, as determined from measurements of excretion of these cations, with BP was non-linear and was most pronounced in persons consuming high-sodium diets, persons with hypertension, and elderly persons [40].

In addition to this, attention has to be paid to differences in salt sensitivity between races. The genetic predisposition to salt sensitivity of BP in blacks is a common concept [41, 42]. In a trial involving only a small number of participants (*n* = 7/group), Luft et al. investigated the effect of different levels of salt consumption (10, 300, 600, 800, 1200, 1500 mmol sodium/day = 0.23, 6.90, 13.80, 18.40, 27.60, 34.50 g sodium/day) on the BP of normotensive black and white subjects. In blacks, sodium intake as low as 800 mmol sodium/day (18.4 g sodium/day) caused significant BP rise compared with the lowest salt intake group (systolic BP 127 ± 1 vs. 113 ± 2 mmHg, diastolic BP 80 ± 4 vs. 68 ± 3 mmHg; *P* < 0.05). In whites, 1200 mmol sodium/day (27.6 g sodium/day) exerted a similar effect [42]. Nonetheless, research data revealed that healthy individuals can tolerate large amounts of salt for short periods of time.

**Fig. 2:**
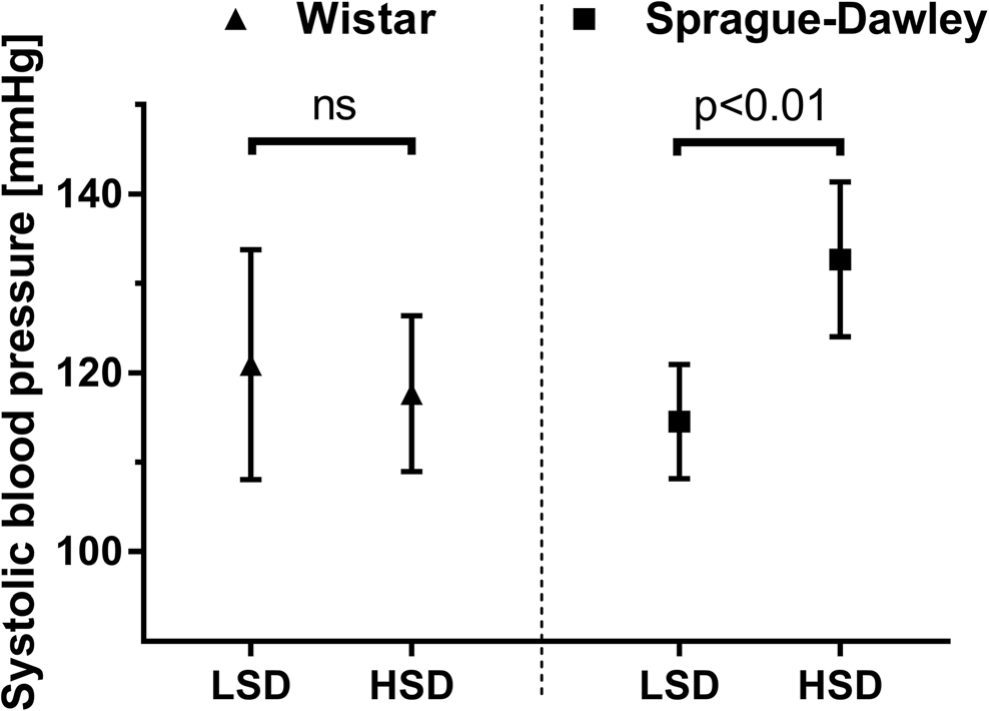
Systolic blood pressure of Wistar (*n* = 8 per group) and Sprague-Dawley (*n* = 11 per group) rats fed on diets with different sodium contents. Blood pressure measurement was performed on vigil animals with a non-invasive tail-cuff blood pressure monitor. LSD (low-salt diet), a sodium-free diet for 4 weeks (< 0.1% salt); HSD (high-salt diet), a diet with 8.0% salt for 4 weeks. Student’s *t* test was performed. Results are shown as mean ± SD (unpublished data). ns, not significant

An animal experiment, involving two rat strains, corroborated the well-known effect of genetic background in salt sensitivity (unpublished data from our research group). High-salt diet after 4 weeks caused systolic BP elevation in salt-sensitive Sprague-Dawley rats (low-salt diet (LSD) 114.6 ± 6.4; high-salt diet (HSD) 132.7 ± 8.7; *P* < 0.01) (Fig. 2). Salt sensitivity turned out to be species-dependent as the BP of Wistar rats receiving the same high-salt diet exhibited no change compared with the low-salt group (LSD 120.9 ± 12.9; HSD 117.7 ± 8.7; *P* > 0.05).

## Salt-sensitive hypertension and special risk groups in children and infants

Only a limited number of clinical trials with focus on children and salt consumption exist. A meta-analysis pointed out that healthy children between the ages of 8 and 16 after a 42% reduction in salt intake exhibited 1.17 mmHg systolic and 1.29 mmHg diastolic BP reduction [43]. For infants, 54% reduction in salt intake yielded a 2.49-mmHg decrease in systolic BP. This means that even in healthy children and infants, salt reduction has a minor, but relevant effect on BP.

An elegant study of Roccini et al. adds important details to the interplay between obesity and salt sensitivity in adolescents [44]. A group of obese and non-obese adolescents were subjected to a salt change diet: 2 weeks of high-salt (> 250 mmol sodium/day = 5.750 g sodium/day) diet was followed by 2 weeks of low-salt (< 30 mmol sodium/day = 0.69 g sodium/day) diet.

After the diet changed, the mean change (± SE) in mean arterial pressure was significantly higher in the obese group (− 12 ± 1 mmHg vs. + 1 ± 2 mmHg; *P* < 0.001). Thereafter, obese participants were enrolled in a 20-week weight loss program and were subjected to the aforementioned dietary high-salt/low-salt protocol. After dietary salt restriction, mean arterial pressure of those participants, who managed to lose a minimum of 1 kg body weight, remained unchanged (mean change ± SE − 1 ± 1 mmHg). In contrast, the BP of participants failed to lose weight were still sensitive to salt intake (mean change ± SE − 11 ± 3 mmHg).

Normal and obese children in the USA were investigated and grouped into quartiles based on their daily sodium intake [45, 46]. Adjusted odds ratios for becoming prehypertensive/hypertensive in the highest sodium intake quartile were 2.0 (95% CI 0.95–4.1, *P* = 0.062) for all participants and 3.5 (95% CI 1.3–9.2, *P* = 0.013) for overweight and obese children compared with those in the respective groups of the lowest sodium intake quartile [46]. This means that especially obese children are at risk of hypertension when consuming a high-salt diet. Sodium intake and weight status appeared to have synergistic effects on the development of hypertension.

Low birth weight seemed to predispose people to hypertension, too [47, 48]. Adult individuals with the history of low birth weight were found to be sensitive to the BP raising effect of salt. For individuals with birth weight under 3050 g, the effect of dietary salt intake on BP was dose-dependent: each 1 g increase in daily salt intake was associated with a 2.48-mmHg higher systolic BP [48]. For individuals with higher birth weight, no significant association between salt intake and systolic or diastolic BP was found.

Low birth weight does not only affect salt sensitivity in adulthood: high dietary salt intake appears to be a modifiable risk factor for intraventricular hemorrhage in low birth weight infants [49].

The salt-sensitizing effect of low birth weight was also seen in an animal experiment [50]: male rat offspring exposed to uteroplacental insufficiency and born small had an increased sensitivity to salt-induced hypertension and arterial remodeling. A chronic salt challenge, even in healthy individuals, would ultimately increase BP. The earlier rise in systolic BP in growth restricted offspring, however, prolonged the exposure time to the negative effects of arterial hypertension which promoted end-organ damage and CV disease.

Not only low birth weight, but also prematurity itself are risk factors of salt sensitivity. In a follow-up experiment, salt sensitivity of BP was demonstrated in preterm-born children at the age of 8 years [51]. This salt sensitivity may contribute to the development of CV disease at later age.

Other perinatal complications including preeclampsia may increase salt sensitivity. Data from an animal experiment showed that in response to a 14-day salt challenge, offspring derived from pregnancies complicated with experimental preeclampsia, compared with offspring from normotensive pregnancies, had a higher systolic BP [52].

## The effect of dietary salt overload on vascular function

This section summarizes the effects salt exerts on the vasculature. Investigations have already revealed that volume overload and/or increased BP secondary to salt overload have detrimental effects on the function of blood vessels’ walls [53]. However, several lines of evidence imply that high salt intake may have adverse impact on the vascular wall—even independently of BP.

In the 1990s, it was observed that following salt overload, the number of arterioles and capillaries in rat skeletal musculature decreases—a phenomenon called microvascular rarefaction [54, 55]. Additionally, the balance of vasoconstrictors and vasodilators gets perturbed at several points: During a diet rich in salt, a higher proportion of 20-hydroxyeicosatetraenoic acid (HETE) is produced, inhibiting hypoxia-induced vasodilatation in mesenteric arterioles [56].

Increased production of the vasoconstrictive end products of cyclooxygenase enzymes contributes to the attenuation of vasorelaxation [57]. Further studies verified the activation of the vascular walls’ local renin-angiotensin system [58]. In contrast to the activation of cyclooxygenase and local renin-angiotensin system, and the intensification of HETE production, the synthesis of the known vasodilator NO gets disturbed; its vasodilator potential decreases significantly [59–61].

With the impairment of the endothelial surface layer (ESL), the condition exacerbates [62]. Sodium-binding glycosaminoglycans (GAGs) are found in large amounts in the ESL of the luminal side of endothelial cells. It has been shown that following high salt consumption, the amount of GAGs and—consequently—the amount of negative charge decrease [62]. Damaged ESL can bring losses of the following functions: (1) water retention due to decreased sodium buffering capacity, (2) shear stress–mediated NO production decreases (3) due to decreased barrier integrity; sodium enters endothelial cells in excess via epithelial sodium channels (ENaC), causing endothelial stiffness.

Most of the above processes manifest themselves in increased peripheral resistance, ultimately leading to high BP and organ damage.

## Extrarenal sodium handling and the impact of sodium on the immune system

Textbook teaching holds that the kidney is the principal organ of water and sodium homeostasis. This notion has been challenged in recent years. Based on long-term sodium balance studies, Titze et al. proposed a new concept of sodium handling [63]. In this model, the excess of dietary sodium intake may bind to the negatively charged GAG molecules of the subcutaneous interstitium of the skin. Sodium stored in this manner may cause local hypertonicity in skin tissue. Infiltrating macrophages sense hypertonicity and promote lymph capillary hyperplasia, which helps temporarily store the excess volume and alleviate BP response to high salt [63].

The presence of sodium deposition in connective tissues was validated using special ^23^Na-MRI measurements, too: age-dependent increase in the amount of stored sodium in the skin was detected [64]. Moreover, it is important to highlight that female patients with refractory hypertension had elevated amounts of stored sodium [65]. Other studies showed an association between increased sodium storage and chronic kidney disease and acute kidney injury [66, 67]. It is uncertain however whether elevated sodium storage is a cause or a consequence of hypertension. The long-term effects of tissue sodium storage on the body are elusive, too.

Salt has multiple—direct and indirect—effects on the immune system. In an in vitro experiment, sodium caused differentiation of T helper 17 (Th17) cells and consequent elevation in the excretion of interleukin-17 (IL-17) [68]. An example for the indirect effect of sodium on Th17 cells is the salt-induced alteration of gut microbiome composition [69]. The relative abundance of salt-sensitive bacteria (e.g. *Lactobacillus* species) following high dietary salt intake decreased in the gut accompanied by an increment in Th17 cells. This implies that metabolic products (butyrate, indole derivatives) of such bacteria are essential for inhibiting the differentiation of T cells into Th17 lineage. Conversely, administration of *Lactobacillus murinus* to experimental animals simultaneously receiving a high-salt diet inhibited IL-17 elevation in the small intestine and the colon. *L*. *murinus* administration exerted an additional anti-hypertensive effect—another piece of evidence, which highlights interconnection between salt consumption and the immune system, and holds promise in the treatment of hypertension [69]. Outside hypertension, Th17 cells have a definitive role in various immune-mediated disorders, too. Several trials tested the impact of salt consumption on immune-mediated disorders [70]. Animal studies established a relationship between dietary sodium intake and the severity of experimental rheumatoid arthritis, multiple sclerosis, and inflammatory bowel diseases [70].

## Conclusions

Despite some methodological shortcomings in estimating/measuring dietary salt intake and some conflicting results, there is plenty of evidence of detrimental effect of high salt intake on human health, particularly cardiovascular disease. But many questions remain: do statistically significant results bear biological relevance? Do we benefit from strict dietary recommendations? In the authors’ view, even healthy adults and children should adhere to international proposals on salt reduction—regardless of how much they benefit from it. According to worldwide surveys, the average of daily salt intake is 10 g with most of it coming from processed food. This highlights the regrettable fact that consumers can barely exercise control over their salt intake. This is particularly important for certain populations who are considered to be salt-sensitive (e.g., African Americans, elderly people, obese people, patients with hypertension, or perinatal complications) and in whom salt restriction would be more likely beneficial from adherence to recommendations. Evidence from randomized controlled interventional studies with an adequately planned methodology are needed to determine the optimal rate of dietary salt intake. For the reasons mentioned above, the question—Is too much salt harmful or not?—should be rephrased as follows: How much and for whom salt intake is harmful?

## Abbreviations

^23^Na-MRI: Sodium magnetic resonance imaging
BP: Blood pressure
CV: Cardiovascular
DASH: Dietary Approaches to Stop Hypertension
ESL: Endothelial surface layer
GAG: Glycosaminoglycan
HETE: Hydroxyeicosatetraenoic acid
IL-17: Interleukin-17
Th17: T helper 17
TOHP: Trial of Hypertension Prevention
WHO: World Health Organization

## References

[1] Denton D, Weisinger R, Mundy NI, Wickings EJ, Dixson A, Moisson P, Pingard AM, Shade R, Carey D, Ardaillou R (1995). The effect of increased salt intake on blood pressure of chimpanzees. Nat Med.

[2] Oliver WJ, Cohen EL, Neel JV (1975). Blood pressure, sodium intake, and sodium related hormones in the Yanomamo Indians, a “no-salt” culture. Circulation.

[3] Powles J, Fahimi S, Micha R, Khatibzadeh S, Shi P, Ezzati M, Engell RE, Lim SS, Danaei G, Mozaffarian D (2013). Global, regional and national sodium intakes in 1990 and 2010: a systematic analysis of 24 h urinary sodium excretion and dietary surveys worldwide. BMJ Open.

[4] Harnack LJ, Cogswell ME, Shikany JM, Gardner CD, Gillespie C, Loria CM, Zhou X, Yuan K, Steffen LM (2017). Sources of sodium in US adults from 3 geographic regions. Circulation.

[5] Bibbins-Domingo K, Chertow GM, Coxson PG, Moran A, Lightwood JM, Pletcher MJ, Goldman L (2010). Projected effect of dietary salt reductions on future cardiovascular disease. N Engl J Med.

[6] WHO guidelines approved by the Guidelines Review Committee (2012). Guideline: sodium intake for adults and children.

[7] Eckel RH, Jakicic JM, Ard JD, Jesus JMD, Miller NH, Hubbard VS, Lee I-M, Lichtenstein AH, Loria CM, Millen BE, Nonas CA, Sacks FM, Sidney C, Smith J, Svetkey LP, Wadden TA, Yanovski SZ (2014) 2013 AHA/ACC guideline on lifestyle management to reduce cardiovascular risk. Circulation. 10.1161/01.cir.0000437740.48606.d1

[8] Appel LJ, Lichtenstein AH, Callahan EA, Sinaiko A, Van Horn L, Whitsel L (2015). Reducing sodium intake in children: a public health investment. J Clin Hypertens (Greenwich).

[9] Elijovich F, Weinberger MH, Anderson CA, Appel LJ, Bursztyn M, Cook NR, Dart RA, Newton-Cheh CH, Sacks FM, Laffer CL (2016). Salt sensitivity of blood pressure: a scientific statement from the American Heart Association. Hypertension (Dallas, Tex : 1979).

[10] Guyton AC (1992). Kidneys and fluids in pressure regulation. Small volume but large pressure changes. Hypertension (Dallas, Tex : 1979).

[11] Guyton AC, Coleman TG, Cowley AV, Scheel KW, Manning RD, Norman RA (1972). Arterial pressure regulation. Overriding dominance of the kidneys in long-term regulation and in hypertension. Am J Med.

[12] Intersalt Cooperative Research Group (1988) Intersalt: an international study of electrolyte excretion and blood pressure. Results for 24 hour urinary sodium and potassium excretion. BMJ 297(6644):319–328 10.1136/bmj.297.6644.319 PMC1834069 3416162

[13] Elliott P, Stamler J, Nichols R, Dyer AR, Stamler R, Kesteloot H, Marmot M (1996). Intersalt revisited: further analyses of 24 hour sodium excretion and blood pressure within and across populations. Intersalt Cooperative Research Group. BMJ.

[14] Hofman A, Hazebroek A, Valkenburg HA (1983). A randomized trial of sodium intake and blood pressure in newborn infants. JAMA.

[15] Geleijnse JM, Hofman A, Witteman JC, Hazebroek AA, Valkenburg HA, Grobbee DE (1997). Long-term effects of neonatal sodium restriction on blood pressure. Hypertension (Dallas, Tex: 1979).

[16] Satterfield S, Cutler JA, Langford HG, Applegate WB, Borhani NO, Brittain E, Cohen JD, Kuller LH, Lasser NL, Oberman A (1991). Trials of hypertension prevention. Phase I design. Ann Epidemiol.

[17] Hebert PR, Bolt RJ, Borhani NO, Cook NR, Cohen JD, Cutler JA, Hollis JF, Kuller LH, Lasser NL, Oberman A (1995). Design of a multicenter trial to evaluate long-term life-style intervention in adults with high-normal blood pressure levels. Trials of Hypertension Prevention (phase II). Trials of Hypertension Prevention (TOHP) Collaborative Research Group. Ann Epidemiol.

[18] (2019) Effects of weight loss and sodium reduction intervention on blood pressure and hypertension incidence in overweight people with high-normal blood pressure: the Trials of Hypertension Prevention, phase II. Arch Intern Med 157(6):657–667. 10.1001/archinte.1997.00440270105009 9080920

[19] Whelton PK, Appel L, Charleston J, Dalcin AT, Ewart C, Fried L, Kaidy D, Klag MJ, Kumanyika S, Steffen L, Walker WG, Oberman A, Counts K, Hataway H, Raczynski J, Rappaport N, Weinsier R, Borhani NO, Bernauer E, Borhani P, Cdl C, Ertl A, Heustis D, Lee M, Lovelace W, O’Connor E, Peel L, Sugars C, Taylor JO, Corkery BW, Evans DA, Keough ME, Morris MC, Pistorino E, Sacks F, Cameron M, Corrigan S, Wright NK, Applegate WB, Brewer A, Goodwin L, Miller S, Murphy J, Randle J, Sullivan J, Lasser NL, Batey DM, Dolan L, Hamill S, Kennedy P, Lasser VI, Kuller LH, Caggiula AW, Milas NC, Yamamoto ME, Vogt TM, Greenlick MR, Hollis J, Stevens V, Cohen JD, Mattfeldt-Beman M, Brinkmann C, Roth K, Shepek L, Hennekens CH, Buring J, Cook N, Danielson E, Eberlein K, Gordon D, Hebert P, MacFadyen J, Mayrent S, Rosner B, Satterfield S, Tosteson H, Denburgh MV, Cutler JA, Brittain E, Farrand M, Kaufmann P, Lakatos E, Obarzanek E, Belcher J, Dommeyer A, Mills I, Neibling P, Woods M, Goldman BJK, Blethen E (2019). The effects of nonpharmacologic interventions on blood pressure of persons with high normal levels: results of the Trials of Hypertension Prevention, phase I. JAMA.

[20] Kalogeropoulos AP, Georgiopoulou VV, Murphy RA, Newman AB, Bauer DC, Harris TB, Yang Z, Applegate WB, Kritchevsky SB (2015). Dietary sodium content, mortality, and risk for cardiovascular events in older adults: the Health, Aging, and Body Composition (Health ABC) Study. JAMA Intern Med.

[21] Taylor RS, Ashton KE, Moxham T, Hooper L, Ebrahim S (2011). Reduced dietary salt for the prevention of cardiovascular disease: a meta-analysis of randomized controlled trials (Cochrane review). Am J Hypertens.

[22] He FJ, Pombo-Rodrigues S, Macgregor GA (2014). Salt reduction in England from 2003 to 2011: its relationship to blood pressure, stroke and ischaemic heart disease mortality. BMJ Open.

[23] Aburto NJ, Ziolkovska A, Hooper L, Elliott P, Cappuccio FP, Meerpohl JJ (2013). Effect of lower sodium intake on health: systematic review and meta-analyses. BMJ.

[24] Strazzullo P, D’Elia L, Kandala NB, Cappuccio FP (2009). Salt intake, stroke, and cardiovascular disease: meta-analysis of prospective studies. BMJ.

[25] Gardener H, Rundek T, Wright CB, Elkind MS, Sacco RL (2012). Dietary sodium and risk of stroke in the Northern Manhattan study. Stroke.

[26] Tomonari T, Fukuda M, Miura T, Mizuno M, Wakamatsu TY, Ichikawa T, Miyagi S, Shirasawa Y, Ito A, Yoshida A, Omori T, Kimura G (2011). Is salt intake an independent risk factor of stroke mortality? Demographic analysis by regions in Japan. J Am Soc Hypertens.

[27] Tuomilehto J, Jousilahti P, Rastenyte D, Moltchanov V, Tanskanen A, Pietinen P, Nissinen A (2001). Urinary sodium excretion and cardiovascular mortality in Finland: a prospective study. Lancet (London, England).

[28] Kapoor K, Fashanu O, Post WS, Lutsey PL, Michos ED, deFilippi CR, McEvoy JW (2019). Relation of dietary sodium intake with subclinical markers of cardiovascular disease (from MESA). Am J Cardiol.

[29] Cook NR, Cutler JA, Obarzanek E, Buring JE, Rexrode KM, Kumanyika SK, Appel LJ, Whelton PK (2007). Long term effects of dietary sodium reduction on cardiovascular disease outcomes: observational follow-up of the trials of hypertension prevention (TOHP). BMJ.

[30] Cook NR, Appel LJ, Whelton PK (2016). Sodium intake and all-cause mortality over 20 years in the Trials of Hypertension Prevention. J Am Coll Cardiol.

[31] Zhao X, Yang X, Zhang X, Li Y, Ren L, Wang L, Gu C, Zhu Z, Han Y (2014). Dietary salt intake and coronary atherosclerosis in patients with prehypertension. J Clin Hypertens (Greenwich).

[32] He J, Ogden LG, Vupputuri S, Bazzano LA, Loria C, Whelton PK (1999). Dietary sodium intake and subsequent risk of cardiovascular disease in overweight adults. JAMA.

[33] Ekinci EI, Clarke S, Thomas MC, Moran JL, Cheong K, MacIsaac RJ, Jerums G (2011). Dietary salt intake and mortality in patients with type 2 diabetes. Diabetes Care.

[34] Mente A, O’Donnell M, Rangarajan S, Dagenais G, Lear S, McQueen M, Diaz R, Avezum A, Lopez-Jaramillo P, Lanas F, Li W, Lu Y, Yi S, Rensheng L, Iqbal R, Mony P, Yusuf R, Yusoff K, Szuba A, Oguz A, Rosengren A, Bahonar A, Yusufali A, Schutte AE, Chifamba J, Mann JF, Anand SS, Teo K, Yusuf S (2016). Associations of urinary sodium excretion with cardiovascular events in individuals with and without hypertension: a pooled analysis of data from four studies. Lancet (London, England).

[35] O’Donnell M, Mente A, Rangarajan S, McQueen MJ, Wang X, Liu L, Yan H, Lee SF, Mony P, Devanath A, Rosengren A, Lopez-Jaramillo P, Diaz R, Avezum A, Lanas F, Yusoff K, Iqbal R, Ilow R, Mohammadifard N, Gulec S, Yusufali AH, Kruger L, Yusuf R, Chifamba J, Kabali C, Dagenais G, Lear SA, Teo K, Yusuf S (2014). Urinary sodium and potassium excretion, mortality, and cardiovascular events. N Engl J Med.

[36] Iatrino R, Manunta P, Zagato L (2016). Salt sensitivity: challenging and controversial phenotype of primary hypertension. Curr Hypertens Rep.

[37] Kempner W (1974). Treatment of hypertensive vascular disease with rice diet. Arch Intern Med.

[38] Sacks FM, Svetkey LP, Vollmer WM, Appel LJ, Bray GA, Harsha D, Obarzanek E, Conlin PR, Miller ER, Simons-Morton DG, Karanja N, Lin PH (2001). Effects on blood pressure of reduced dietary sodium and the Dietary Approaches to Stop Hypertension (DASH) diet. DASH-Sodium Collaborative Research Group. N Engl J Med.

[39] He FJ, Li J, Macgregor GA (2013). Effect of longer term modest salt reduction on blood pressure: Cochrane systematic review and meta-analysis of randomised trials. BMJ.

[40] Mente A, O’Donnell MJ, Rangarajan S, McQueen MJ, Poirier P, Wielgosz A, Morrison H, Li W, Wang X, Di C, Mony P, Devanath A, Rosengren A, Oguz A, Zatonska K, Yusufali AH, Lopez-Jaramillo P, Avezum A, Ismail N, Lanas F, Puoane T, Diaz R, Kelishadi R, Iqbal R, Yusuf R, Chifamba J, Khatib R, Teo K, Yusuf S (2014). Association of urinary sodium and potassium excretion with blood pressure. N Engl J Med.

[41] Falkner B, Kushner H (1990). Effect of chronic sodium loading on cardiovascular response in young blacks and whites. Hypertension (Dallas, Tex : 1979).

[42] Luft FC, Rankin LI, Bloch R, Weyman AE, Willis LR, Murray RH, Grim CE, Weinberger MH (1979). Cardiovascular and humoral responses to extremes of sodium intake in normal black and white men. Circulation.

[43] He FJ, MacGregor GA (2006). Importance of salt in determining blood pressure in children: meta-analysis of controlled trials. Hypertension (Dallas, Tex : 1979).

[44] Rocchini AP, Key J, Bondie D, Chico R, Moorehead C, Katch V, Martin M (1989). The effect of weight loss on the sensitivity of blood pressure to sodium in obese adolescents. N Engl J Med.

[45] Lava SA, Bianchetti MG, Simonetti GD (2015). Salt intake in children and its consequences on blood pressure. Pediatr Nephrol (Berlin, Germany).

[46] Yang Q, Zhang Z, Kuklina EV, Fang J, Ayala C, Hong Y, Loustalot F, Dai S, Gunn JP, Tian N, Cogswell ME, Merritt R (2012). Sodium intake and blood pressure among US children and adolescents. Pediatrics.

[47] de Boer MP, Ijzerman RG, de Jongh RT, Eringa EC, Stehouwer CD, Smulders YM, Serne EH (2008). Birth weight relates to salt sensitivity of blood pressure in healthy adults. Hypertension (Dallas, Tex : 1979).

[48] Perala MM, Moltchanova E, Kaartinen NE, Mannisto S, Kajantie E, Osmond C, Barker DJ, Valsta LM, Eriksson JG (2011). The association between salt intake and adult systolic blood pressure is modified by birth weight. Am J Clin Nutr.

[49] Barnette AR, Myers BJ, Berg CS, Inder TE (2010). Sodium intake and intraventricular hemorrhage in the preterm infant. Ann Neurol.

[50] Gallo LA, Walton SL, Mazzuca MQ, Tare M, Parkington HC, Wlodek ME, Moritz KM (2018). Uteroplacental insufficiency temporally exacerbates salt-induced hypertension associated with a reduced natriuretic response in male rat offspring. J Physiol.

[51] Ruys CA, Rotteveel J, van de Lagemaat M, Lafeber HN, Finken MJJ (2018). Salt sensitivity of blood pressure at age 8 years in children born preterm. J Hum Hypertens.

[52] Yeung KR, Sunderland N, Lind JM, Heffernan S, Pears S, Xu B, Hennessy A, Makris A (2018). Increased salt sensitivity in offspring of pregnancies complicated by experimental preeclampsia. Clin Exp Pharmacol Physiol.

[53] Dumont O, Pinaud F, Guihot AL, Baufreton C, Loufrani L, Henrion D (2008). Alteration in flow (shear stress)-induced remodelling in rat resistance arteries with aging: improvement by a treatment with hydralazine. Cardiovasc Res.

[54] Hansen-Smith FM, Morris LW, Greene AS, Lombard JH (1996). Rapid microvessel rarefaction with elevated salt intake and reduced renal mass hypertension in rats. Circ Res.

[55] Hernandez I, Cowley AW, Lombard JH, Greene AS (1992). Salt intake and angiotensin II alter microvessel density in the cremaster muscle of normal rats. Am J Phys.

[56] Wang J, Roman RJ, Falck JR, de la Cruz L, Lombard JH (2005). Effects of high-salt diet on CYP450-4A omega-hydroxylase expression and active tone in mesenteric resistance arteries. Am J Phys Heart Circ Phys.

[57] Cavka A, Cosic A, Jukic I, Jelakovic B, Lombard JH, Phillips SA, Seric V, Mihaljevic I, Drenjancevic I (2015). The role of cyclo-oxygenase-1 in high-salt diet-induced microvascular dysfunction in humans. J Physiol.

[58] Boddi M, Poggesi L, Coppo M, Zarone N, Sacchi S, Tania C, Neri Serneri GG (1998). Human vascular renin-angiotensin system and its functional changes in relation to different sodium intakes. Hypertension (Dallas, Tex : 1979).

[59] Cavka A, Jukic I, Ali M, Goslawski M, Bian JT, Wang E, Drenjancevic I, Phillips SA (2016). Short-term high salt intake reduces brachial artery and microvascular function in the absence of changes in blood pressure. J Hypertens.

[60] Greaney JL, DuPont JJ, Lennon-Edwards SL, Sanders PW, Edwards DG, Farquhar WB (2012). Dietary sodium loading impairs microvascular function independent of blood pressure in humans: role of oxidative stress. J Physiol.

[61] Zhu J, Mori T, Huang T, Lombard JH (2004). Effect of high-salt diet on NO release and superoxide production in rat aorta. Am J Phys Heart Circ Phys.

[62] Olde Engberink RH, Rorije NM, Homan van der Heide JJ, van den Born BJ, Vogt L (2015). Role of the vascular wall in sodium homeostasis and salt sensitivity. J Am Soc Nephrol.

[63] Machnik A, Neuhofer W, Jantsch J, Dahlmann A, Tammela T, Machura K, Park JK, Beck FX, Muller DN, Derer W, Goss J, Ziomber A, Dietsch P, Wagner H, van Rooijen N, Kurtz A, Hilgers KF, Alitalo K, Eckardt KU, Luft FC, Kerjaschki D, Titze J (2009). Macrophages regulate salt-dependent volume and blood pressure by a vascular endothelial growth factor-C-dependent buffering mechanism. Nat Med.

[64] Linz P, Santoro D, Renz W, Rieger J, Ruehle A, Ruff J, Deimling M, Rakova N, Muller DN, Luft FC, Titze J, Niendorf T (2015). Skin sodium measured with (2)(3)Na MRI at 7.0 T. NMR Biomed.

[65] Kopp C, Linz P, Dahlmann A, Hammon M, Jantsch J, Muller DN, Schmieder RE, Cavallaro A, Eckardt KU, Uder M, Luft FC, Titze J (2013). 23Na magnetic resonance imaging-determined tissue sodium in healthy subjects and hypertensive patients. Hypertension (Dallas, Tex : 1979).

[66] Dahlmann A, Dorfelt K, Eicher F, Linz P, Kopp C, Mossinger I, Horn S, Buschges-Seraphin B, Wabel P, Hammon M, Cavallaro A, Eckardt KU, Kotanko P, Levin NW, Johannes B, Uder M, Luft FC, Muller DN, Titze JM (2015). Magnetic resonance-determined sodium removal from tissue stores in hemodialysis patients. Kidney Int.

[67] Hammon M, Grossmann S, Linz P, Seuss H, Hammon R, Rosenhauer D, Janka R, Cavallaro A, Luft FC, Titze J, Uder M, Dahlmann A (2017). 3 tesla 23Na magnetic resonance imaging during acute kidney injury. Acad Radiol.

[68] Kleinewietfeld M, Manzel A, Titze J, Kvakan H, Yosef N, Linker RA, Muller DN, Hafler DA (2013). Sodium chloride drives autoimmune disease by the induction of pathogenic TH17 cells. Nature.

[69] Wilck N, Matus MG, Kearney SM, Olesen SW, Forslund K, Bartolomaeus H, Haase S, Mahler A, Balogh A, Marko L, Vvedenskaya O, Kleiner FH, Tsvetkov D, Klug L, Costea PI, Sunagawa S, Maier L, Rakova N, Schatz V, Neubert P, Fratzer C, Krannich A, Gollasch M, Grohme DA, Corte-Real BF, Gerlach RG, Basic M, Typas A, Wu C, Titze JM, Jantsch J, Boschmann M, Dechend R, Kleinewietfeld M, Kempa S, Bork P, Linker RA, Alm EJ, Muller DN (2017). Salt-responsive gut commensal modulates TH17 axis and disease. Nature.

[70] Toussirot E, Bereau M, Vauchy C, Saas P (2018). Could sodium chloride be an environmental trigger for immune-mediated diseases? An overview of the experimental and clinical evidence. Front Physiol.

